# Phase II Trial of Dolastatin-10, a Novel Anti-Tubulin Agent, in Metastatic Soft Tissue Sarcomas

**DOI:** 10.1155/2004/924913

**Published:** 2004-12-22

**Authors:** M. von Mehren, S. P. Balcerzak, A. S. Kraft, J. H. Edmonson, S. H. Okuno, M. Davey, S. Mclaughlin, M. T. Beard, A. Rogatko

**Affiliations:** ^1^ Department of Medical Oncology, Fox Chase Cancer Center, Philadelphia, PA 19111, USA; ^2^ Ohio State University, OH, USA; ^3^ University of Colorado, CO, USA; ^4^ Mayo Clinic, Emory University, Atlanta, GA, USA; ^5^ Department of Biostatistics, Emory University, Atlanta, GA, USA

## Abstract

*Patients*: Soft tissue sarcomas are uncommon malignancies with few therapeutic options for recurrent or metastatic disease. Dolastatin-10 (Dol-10) is a pentapeptide anti-microtubule agent that binds to tubulin sites distinct from vinca alkaloids. Based on the novel mechanism of action, limited activity of other anti-microtubular agents, and anti-neoplastic activity in pre-clinical screening of Dol-10, this multi-institutional phase II study was conducted to determine the objective response rate of Dol-10 in recurrent or metastatic soft tissue sarcomas that had not been treated with chemotherapy outside of the adjuvant setting.

*Methods*: Dol-10 was given intravenously at a dose of 400 μg/m2 and repeated every 21 days. Toxicities were assessed using the Common Toxicity Criteria (version 2.0). Radiographic studies and tumor measurements were repeated every two cycles to assess response [Miller AB, et al. Cancer 1981; 47(1): 207].

*Results*: Dol-10 was associated with hematological toxicity and with some vascular toxicities. There was no significant gastrointestinal, hepatic or renal toxicity. There was one death on study due to respiratory failure. There were no objective responses in 12 patients treated with Dol-10.

*Discussion*: Based on this phase II trial, further study of Dol-10 on this schedule is not recommended in advanced or metastatic soft tissue sarcomas.


*Sarcoma*, December 2004, *VOL. 8, NO. 4, 107–111*

Taylor & Francis healthsciences

ORIGINAL ARTICLE

### Phase II trial of dolastatin-10, a novel anti-tubulin agent, in metastatic soft tissue sarcomas

M. VON MEHREN[1], S.P. BALCERZAK[2], A.S. KRAFT[3], J.H. EDMONSON[4], S.H. OKUNO[4], M. DAVEY[1], S. McLAUGHLIN[1], M.T. BEARD[1] & A. ROGATKO[5]

[1]*Department of Medical Oncology, Fox Chase Cancer Center, Philadelphia, PA 19111, USA,* [2]*Ohio State University, OH,* [3]*University of Colorado, CO,* [4]*Mayo Clinic,* [5]*Department of Biostatistics, Emory University, Atlanta, GA, USA*

**Abstract**

*Patients:* Soft tissue sarcomas are uncommon malignancies with few therapeutic options for recurrent or metastatic disease. Dolastatin-10 (Dol-10) is a pentapeptide anti-microtubule agent that binds to tubulin sites distinct from vinca alkaloids. Based on the novel mechanism of action, limited activity of other anti-microtubular agents, and anti-neoplastic activity in pre-clinical screening of Dol-10, this multi-institutional phase II study was conducted to determine the objective response rate of Dol-10 in recurrent or metastatic soft tissue sarcomas that had not been treated with chemotherapy outside of the adjuvant setting.

*Methods:* Dol-10 was given intravenously at a dose of 400 μg/m$^2$ and repeated every 21 days. Toxicities were assessed using the Common Toxicity Criteria (version 2.0). Radiographic studies and tumor measurements were repeated every two cycles to assess response [Miller AB, et al. *Cancer* 1981; 47(1): 207].

*Results:* Dol-10 was associated with hematological toxicity and with some vascular toxicities. There was no significant gastrointestinal, hepatic or renal toxicity. There was one death on study due to respiratory failure. There were no objective responses in 12 patients treated with Dol-10.

*Discussion:* Based on this phase II trial, further study of Dol-10 on this schedule is not recommended in advanced or metastatic soft tissue sarcomas.

**Key words:** *dolastatin-10, anti-microtubule agent, soft tissue sarcoma*

### Abbreviation

| | |
|---|---|
| Dol-10 | dolastatin-10 |
| STS | soft tissue sarcomas |
| ip | intraperitoneal |
| DAB | diaminobenzidene |
| GIST | gastrointestinal stromal tumor |

### Introduction

Soft tissue sarcomas (STS) are an uncommon tumor with poor survival. Some patients with metastatic or locally recurrent disease require chemotherapy and radiation therapy for palliation. In the metastatic disease setting, chemotherapy regimens have an overall response rate of 30–40%, with only rare prolonged complete remissions. Clinical trials of doxorubicin as a single agent or in combined modality therapies have demonstrated response rates in the range of 15–34%, although without evidence for a survival benefit.[2–5] The anti-tumor activity of other chemotherapeutic agents,[6–10] high dose therapy with transplantation,[11,12] and immunological therapies[13–16] have not been encouraging. Consequently, there is an urgent need to identify new active agents for this disease. Given the limited activity of agents available for the treatment of soft tissue sarcoma, a trial testing a new agent as first line therapy was considered acceptable.

Dolastatins are naturally occurring pentapeptides isolated from the East Indian Ocean Sea hare *Dolabella auricularia.*[17] Extracts from the sea hare were found to have anti-tumor activity in the NCI intraperitoneal (ip) P388 leukemia model. Dol-10, which is now synthesized,[18] has a linear peptide structure of four amino acids linked to a complex primary amine. Three of its amino acids (dolavaline, dolaisoleucine, and dolaproline) and its terminal amine (dolaphenine) are unique to the mollusk from which it was isolated.[19] Dol-10 is a potent antimotic agent, similar to the vinca alkaloids and taxanes.

Correspondence to: Margaret von Mehren, Fox Chase Cancer Center, Philadelphia, PA 19111, USA. Tel.: +1-215-728-3545; Fax: +1-215-728-3639; E-mail: m_vonmehren@fccc.edu

1357-714X print/1369–1643 online © 2004 Taylor & Francis Ltd
DOI: 10.1080/13577140400009163

However, studies suggest it binds to $\beta$-tubulin at a site distinct from the vinca alkaloid binding site.[17,20] The anti-microtubule agents vindesine, paclitaxel and docetaxel have been investigated in STS with response rates up to 19% in phase II studies.[21–23] Thus, in STS it is reasonable to evaluate novel agents in this class that bind to unique sites on tubulin. The purpose of this multi-institutional phase II study was to evaluate the toxicity and efficacy of Dol-10 in patients with recurrent or metastatic disease, not previously treated with chemotherapy outside of the adjuvant setting.

### Patients

This multi-institutional study enrolled patients at Fox Chase Cancer Center, Mayo Clinic, Ohio State University, and University of Colorado between April 1999 and February 2000. Eligibility criteria included: written informed consent, histologically confirmed soft tissue sarcoma that was recurrent or metastatic, measurable disease, no prior chemotherapy except adjuvant chemotherapy, no prior radiation therapy unless there was progressive measurable disease outside of the radiation port, an Eastern Cooperative Oncology Group performance status of 0 or 1, and adequate hepatic, renal, and bone marrow function. Women of child-bearing potential had to have a negative pregnancy test within 72 h prior to the first cycle. Patients were required to use contraception during study participation.

Exclusion criteria included: a diagnosis of rhabdomyosarcoma, Ewing's sarcoma, chondrosarcoma, osteogenic sarcoma, mesothelioma, or Kaposi's sarcoma; also a history of brain metastases, uncontrolled infection, breast-feeding female patients or a history of malignancy other than basal or squamous cell carcinomas of the skin, or *in situ* carcinoma of the cervix, diagnosed within the past 5 years.

### Materials and Methods

### *Protocol procedures*

Dol-10 was supplied by the Cancer Therapeutics Evaluation Program of the National Cancer Institute. Patients received Dol-10, 400 $\mu g/m^2$ intravenously over 10 min every 21 days. Complete blood counts were obtained weekly and a chemistry panel every 3 weeks. Toxicities were assessed using the Common Toxicity Criteria (version 2.0). Tumor measurements were repeated every two cycles to assess response using standard criteria.[1]

### *Dose modification criteria*

Grade 4 hematological toxicity at any time resulted in a 25% dose reduction. If tolerated without a further grade 3–4 toxicity, one dose escalation of 12.5% was allowed. Patients with grade 3–4 hematological toxicity on day 21 were delayed 7 days. If not recovered after 7 days, patients were taken off study. Any other non-hematological or neurological grade 3–4 toxicity required a 25% dose reduction, with the exception of nausea, vomiting, and diarrhea, unless inadequately controlled by optimal supportive therapies. Patients who tolerated reduced doses were allowed a 12.5% (for prior grade 4) and 25% (for prior grade 3) dose escalation, whereas those whose toxicity did not improve to less than a grade 3 were taken off study. Patients who received at least 2 consecutive cycles of 400 $\mu gm/m^2$ without grade 3–4 toxicities were eligible for a dose escalation to 450 $\mu gm/m^2$.

### *Statistical analysis*

This phase II study was designed to assess the frequency and extent of anti-tumor activity of Dol-10 in a population of patients with recurrent or metastatic soft tissue sarcoma. The primary endpoint was overall objective response proportion (complete and partial response). At the time this trial was designed, a proportion of patients with favorable response less than 5.0% was deemed of no interest. The new treatment would be of interest if the proportion of patients ($p$) with tumor response was at least 20.0%. Thirty-seven patients would be needed to test the null hypothesis ($P \leq 0.050$) against the alternative hypothesis ($p \geq 0.200$) at the 3.2% level of significance and with 84.8% power. An early stopping rule for futility was applied such that if no patients with CR or PR were observed when 12 patients were accrued, then the null hypothesis would be accepted and the trial terminated. The probability of early stopping under the null hypothesis was 0.54, and under the alternative was 0.07. If the trial progressed until 37 patients were evaluated and five or more patients with favorable response were observed, then the null hypothesis would be rejected.

### Results

### *Patient characteristics*

A total of twelve patients were enrolled on the study from April 1999 to February 2000 at the four centers. Patient characteristics are summarized in Table 1. The most common diagnosis was leiomyosarcoma. Pretreatment was minimal with only one patient treated with chemotherapy in the adjuvant setting. Four patients had prior adjuvant radiation therapy and one had palliative therapy. A total of 26 cycles of treatment were given. One patient received five cycles, 10 patients received 2 cycles, and one patient received only one cycle of therapy.

**Table 1.** *Patient characteristics*

| | N (%) |
|---|---|
| *Age* | |
| Range | 40–74 |
| Median | 57 |
| *Sex* | |
| Male | 4 (33%) |
| Female | 8 (67%) |
| *Performance status* | |
| PS=0 | 5 (42%) |
| PS=1 | 7 (58%) |
| *Histology* | |
| Leiomyosarcoma | 7 (58%)* |
| Synovial sarcoma | 2 (17%) |
| Malignant fibrous histiocytoma | 1 (8%) |
| Angiosarcoma | 1 (8%) |
| Spindle Cell | 1 (8%) |
| *Prior therapy* | |
| Surgery | 12 (100%) |
| Radiation therapy | 4 (33%) |
| Adjuvant chemotherapy | 1 (8%) |

*Following trial participation, one patient was found to have KIT expression and was reclassified as having a gastrointestinal stromal tumor.

**Table 2.** *Summary of toxicity using the Common Toxicity Criteria version 2.0: worst grade for all cycles of treatment*

| Toxicity | Grade and No. of patients with toxicity | | | |
|---|---|---|---|---|
| | 1 | 2 | 3 | 4 |
| White blood cell | 2 | 1 | 3 | 0 |
| Neutrophil count | 0 | 0 | 3 | 1 |
| Platelet count | 2 | 0 | 0 | 0 |
| Anemia | 10 | 1 | 2 | 0 |
| Bleeding | 0 | 0 | 1 | 1 |
| Abdominal pain | 1 | 1 | 1 | 0 |
| Nausea | 2 | 2 | 0 | 0 |
| Vomiting | 0 | 3 | 0 | 0 |
| Liver function | 4 | 1 | 0 | 0 |
| Hyponatremia | 2 | 0 | 1 | 0 |
| Hypoalbuminemia | 5 | 0 | 0 | 0 |
| Hypokalemia | 2 | 0 | 1 | 0 |
| Edema | 0 | 0 | 2 | 0 |
| Dizziness | 1 | 0 | 2 | 0 |
| Hypotension | 1 | 0 | 1 | 0 |
| Hypertension | 1 | 0 | 1 | 0 |
| Arrhythmia | 2 | 0 | 2 | 0 |
| Chest Pain | 1 | 1 | 0 | 0 |
| SOB/hypoxia | 0 | 1 | 1 | 2* |
| Fatigue/weakness | 12 | 2 | 2 | 1 |
| Headache | 2 | 0 | 1 | 0 |
| Night sweats | 2 | 1 | 0 | 0 |
| Skin changes | 2 | 0 | 0 | 0 |
| IV site reactions | 1 | 3 | 0 | 0 |
| Sacral pain | 0 | 2 | 0 | 0 |

Toxicity observed in all cycles of Dol-10 using the Common Toxicity Criteria, version 2.0.
*One event resulted in death within 30 days of drug and is classified as a death on study.

### *Toxicity*

Table 2 describes the toxicities observed during all 26 cycles of therapy. The majority of the observed adverse events were grade 1 or 2 (71%). Anemia was the most frequent hematological toxicity. Grade 3 anemia was noted in a patient with leiomyosarcoma and a history of intraperitoneal bleeding who presented on cycle 1, day 2 with abdominal pain and hypotension. CT scan revealed hemoperitoneum. After cycle 2 with a 25% dose reduction, the patient had recurrent hemoperitoneum with bleeding from tumor confirmed at exploratory laporotamy. The recurrent episodes in a tumor known for bleeding implicated the underlying disease rather than Dol-10 as the likely cause. The second patient with grade 3 anemia, also with leiomyosarcoma, began treatment with grade one anemia and had a progressive decline in hemoglobin without evidence of bleeding.

There were three episodes of grade 3 leukopenia associated with concurrent neutropenia. Nadirs were seen on day 21, requiring a week delay. Only one patient developed an *Eschericiae coli* urinary tract infection in association with grade 4 neutropenia. Alterations in liver function tests were all associated with progressive metastatic liver disease at the initial response evaluation.

Two patients experienced grade 3 and 4 cardiopulmonary toxicities, with one resulting in death. Patient 2 had a history of spindle cell sarcoma with a past medical history significant for hypertension. On day 1 she was noted to be hypertensive having not taken her blood pressure medications. Her blood pressure increased after the Dol-10 infusion, but improved with anti-hypertensive therapy. On day 2 the patient had persistent hypertension, and her anti-hypertensive medications were increased. She stopped taking her anti-hypertensives again on day 4 and was hospitalized on day 8 with hypertension and congestive heart failure. She expired on day 10 of respiratory failure. As hypertension has been reported as a potential side effect of dolastatins, this was considered to be a possible drug toxicity. The second patient had tachycardia secondary to hemoperitoneum and anemia as described above.

### *Clinical response*

Of the 12 patients entered on study, 11 received at least two cycles of therapy. Ten patients were removed from study after the first two cycles of therapy with progressive disease. Patient 12 received five cycles of therapy. Radiographically the patient had stable disease but had increasing tumor related pain and was removed from study to receive palliative radiation therapy.

### Discussion

STS are a heterogeneous group of tumors with few effective therapies. This study evaluated a new anti-microtubular agent in patients with recurrent or metastatic disease who were chemotherapy naïve with the exception of adjuvant therapy. Because there were no objective responses in 12 evaluable patients, the study was closed to further accrual. The heterogeneity of histological STS subtypes may have obscured activity in one histological subset as too few patients of any one subtype were treated.

In preclinical studies, Dol-10 was active against leukemia,[24] and non-small cell lung cancer cell lines.[25] Phase I and phase II studies utilizing Dol-10 did not demonstrate any objective responses in a variety of solid tumors.[26] A trial in acute leukemia demonstrated decreases in peripheral blast counts, and one patient had a partial response.[27] The toxicities observed in our patients were consistent with those observed in phase I studies of Dol-10. The hypertension noted in this study was described preclinically in dog studies and in one prior phase I trial.[28] Another compound derived from *Dolabela auricularia*, Dolastatin-15, had arterial hypertension and myocardial infarction as the dose-limiting toxicities.

Among the possibilities for the lack of efficacy of Dol-10 is inherent in phase II trials of soft tissue sarcomas due to the multiple histologies accrued to studies. In addition, review of patient histories suggests that four of the seven patients with leiomyosarcoma likely had gastrointestinal stromal tumor (GIST), with one confirmed by KIT immunostaining. This histology has been shown to be refractory to chemotherapy.[29] These data were not available at the time of the development of this study. If our study contained four GIST patients out of the 12 patients, this may in part account for the lack of response seen. Another potential etiology for the inactivity is multidrug resistance, as STS are known to express high levels of MDR1.[30] Murine and human leukemia cell lines that express MDR1 have been shown to be resistant to Dol-10.[31] In addition, a clinical trial in acute leukemias in which patients were noted to have a decline in their peripheral blast count, suggested a correlation between Dol-10 efficacy and MDR1 expression.[27]

In summary, as gauged by standard response criteria, Dol-10 as first-line therapy was inactive in 12 patients with recurrent or metastatic soft tissue sarcoma. The lack of efficacy is particularly striking in this patient population with minimal pre-treatment. Recent data from the European Organization for Research and Treatment of Cancer Soft Tissue and Bone Sarcoma Group has identified therapy with doxorubicin-based regimens as a factor associated with improved long-term survival in patients with metastatic soft tissue sarcoma.[32] Future phase II studies should consider the appropriateness of testing new agents in patients that have not previously been treated with doxorubicin.

### Acknowledgments

We acknowledge Dr John Wright of the Cancer Therapeutics Evaluation Program of the National Cancer Center for his assistance with this trial. In addition, we acknowledge Dr Gary Hudes for his aid in developing the protocol and in thoughtful review of the manuscript.

### References

1. Miller AB, Hoogstraten B, Staquet M, Winkler A. Reporting results of cancer treatment. *Cancer* 1981; 47(1): 207–14.
2. Borden EC, Amato DA, Rosenbaum C, *et al.* Randomized comparison of three adriamycin regimens for metastatic soft tissue sarcomas. *J Clin Oncol* 1987; 5(6): 840–50.
3. O'Bryan RM, Baker LH, Gottlieb JE, *et al.* Dose response evaluation of adriamycin in human neoplasia. *Cancer* 1977; 39(5): 1940–8.
4. Omura GA, Major FJ, Blessing JA, *et al.* A randomized study of adriamycin with and without dimethyl triazenoimidazole carboxamide in advanced uterine sarcomas. *Cancer* 1983; 52(4): 626–32.
5. Elias A, Ryan L, Aisner J, Antman KH. Mesna, doxorubicin, ifosfamide, dacarbazine (MAID) regimen for adults with advanced sarcoma. *Semin Oncol* 1990; 17(2 Suppl 4): 41–9.
6. Casper ES, Christman KL, Schwartz GK, Johnson B, Brennan MF, Bertino JR. Edatrexate in patients with soft tissue sarcoma. Activity in malignant fibrous histiocytoma. *Cancer* 1993; 72(3): 766–70.
7. Wagener DJ, Somers R, Santoro A, *et al.* Phase II study of nimustine in metastatic soft tissue sarcoma. European Organization for Research and Treatment of Cancer Soft Tissue and Bone Sarcoma Group. *Eur J Cancer* 1991; 27(12): 1604–5.
8. Roche H, Guiochet N, Kerbrat P, *et al.* Phase II trials of tetrahydropyranyl-adriamycin (Pirarubicin) on renal and colon carcinoma, melanoma, and soft tissue sarcoma. *Am J Clin Oncol* 1993; 16(2): 137–9.
9. Zalupski MM, Benedetti J, Balcerzak SP, *et al.* Phase II trial of piroxantrone for advanced or metastatic soft tissue sarcomas. A Southwest Oncology Group study. *Invest New Drugs* 1993; 11(4): 337–41.
10. Licht JD, Gonin R, Antman KH. Phase II trial of trimetrexate in patients with advanced soft-tissue sarcoma. *Cancer Chemother Pharmacol* 1991; 28(3): 223–5.
11. Bokemeyer C, Franzke A, Hartmann JT, *et al.* A phase I/II study of sequential, dose-escalated, high dose ifosfamide plus doxorubicin with peripheral blood stem cell support for the treatment of patients with advanced soft tissue sarcomas. *Cancer* 1997; 80(7): 1221–7.
12. Blay JY, Bouhour D, Ray-Coquard I, Dumontet C, Philip T, Biron P. High-dose chemotherapy with autologous hematopoietic stem-cell transplantation for advanced soft tissue sarcoma in adults. *J Clin Oncol* 2000; 18(21): 3643–50.
13. Le Cesne A, Vassal G, Farace F, *et al.* Combination interleukin-2 and doxorubicin in advanced adult solid tumors: circumvention of doxorubicin resistance in soft-tissue sarcoma? *J Immunother* 1999; 22(3): 268–77.

14. Galanis E, Hersh E, Stopeck A, *et al.* Immunotherapy of advanced malignancy by direct gene transfer of an interleukin-2 DNA/DMRIE/DOPE lipid complex: phase I/II experience. *J Clin Oncol* 1999; 17: 3313–23.
15. Gold JE, Masters TR, Bloom ND, *et al.* Ex vivo activated memory T-lymphocytes as adoptive cellular therapy of human soft-tissue sarcoma targets with potentiation by cis-diamminedichloroplatinum(II). *J Surg Oncol* 1995; 58(4): 212–21.
16. Casper ES, Cordon-Cardo C, Houghton AN, *et al.* Biological study of R24 mouse monoclonal antibody in patients undergoing thoracotomy for pulmonary metastases from soft tissue sarcoma. *Cancer Invest* 1994; 12(1): 20–5.
17. Bai R, Pettit GR, Hamel E. Dolastatin 10, a powerful cytostatic peptide derived from a marine animal. Inhibition of tubulin polymerization mediated through the vinca alkaloid binding domain. *Biochem Pharmacol* 1990; 39(12): 1941–9.
18. Bai R, Roach MC, Jayaram SK, *et al.* Differential effects of active isomers, segments, and analogs of dolastatin 10 on ligand interactions with tubulin. Correlation with cytotoxicity. *Biochem Pharmacol* 1993; 45(7): 1503–15.
19. Hamel E. Natural products which interact with tubulin in the vinca domain: maytansine, rhizoxin, phomopsin A, dolastatins 10 and 15 and halichondrin B. *Pharmacol Ther* 1992; 55(1): 31–51.
20. Bai R, Taylor GF, Cichacz ZA, *et al.* The spongistatins, potently cytotoxic inhibitors of tubulin polymerization, bind in a distinct region of the vinca domain. *Biochemistry* 1995; 34(30): 9714–21.
21. Borden EC, Amato DA, Edmonson JH, Ritch PS, Shiraki M. Randomized comparison of doxorubicin and vindesine to doxorubicin for patients with metastatic soft-tissue sarcomas. *Cancer* 1990; 66(5): 862–7.
22. Balcerzak SP, Benedetti J, Weiss GR, Natale RB. A phase II trial of paclitaxel in patients with advanced soft tissue sarcomas. A Southwest Oncology Group study. *Cancer* 1995; 76(11): 2248–52.
23. van Hoesel QG, Verweij J, Catimel G, *et al.* Phase II study with docetaxel (Taxotere) in advanced soft tissue sarcomas of the adult. EORTC Soft Tissue and Bone Sarcoma Group. *Ann Oncol* 1994; 5(6): 539–42.
24. Petit G, Kamano Y, Herald C, *et al.* The isolation and structure of a remarkable marine animal antineoplastic constituent: dolastatin 10. *J Am Chem Soc* 1987; 109: 6883–5.
25. Kalemkerian GP, Ou X, Adil MR, *et al.* Activity of dolastatin 10 against small-cell lung cancer *in vitro* and *in vivo*: induction of apoptosis and bcl-2 modification. *Cancer Chemother Pharmacol* 1999; 43(6): 507–15.
26. Hoffman M, Blessing J, Lentz S. A phase II trial of dolastatin-10 in recurrent platinum-sensitive ovarian carcinoma: a Gynecologic Oncology Group study. *Gynecol Oncol* 2003; 89: 95–8.
27. Cheson B, Zwiebel J, Dancey J, Murgo A. Novel Therapeutic agents for the treatment of myelodysplastic syndromes. *Semin Oncol* 2000; 27(5): 560–77.
28. Pitot H, McElroy E, Reid J, *et al.* Phase I trial of dolastatin-10 (NSC 376128) in patients with advanced solid tumors. *Clin Cancer Res* 1999; 5: 525–31.
29. van Glabbeke M, Verweij J, Judson I, Nielsen OS. EORTC Soft Tissue and Bone Sarcoma Group. Progression-free rate as the principal end-point for phase II trials in soft-tissue sarcomas. *Eur J Cancer* 2002; 38: 543–9.
30. Plaat BEC, Hollema H, Molenaar WM, *et al.* Soft tissue leiomyosarcomas and malignant gastrointestinal stromal tumors: differences in clinical outcome and expression of multidrug resistance proteins. *J Clin Oncol* 2000; 18(18): 3211–20.
31. Toppmeyer DL, Slapak CA, Croop J, Kufe DW. Role of P-glycoprotein in dolastatin 10 resistance. *Biochem Pharmacol* 1994; 48(3): 609–12.
32. Blay J, van Glabbeke M, Verweij J, *et al.* Advanced soft-tissue sarcoma: a disease that is potentially curable for a subset of patients treated with chemotherapy. *Eur J Cancer* 2003; 39: 64–9.
